# Resistance to age-related hypercoagulability: insights from the naked mole rat

**DOI:** 10.1016/j.rpth.2026.103458

**Published:** 2026-03-26

**Authors:** Véronique Regnault, Jeremy Lagrange, Chris G. Faulkes, J. Kennedy Cruickshank, Cécile Lakomy, Athanase Benetos, Cécile V. Denis, Patrick Lacolley

**Affiliations:** 1 Université de Lorraine, INSERM, DCAC, Nancy, France; 2 School of Biological and Chemical Sciences, Queen Mary University of London, London, United Kingdom; 3 Cardiovascular Medicine-Diabetes Group, School of Life Course-Nutritional Sciences Division, King’s College London, London, United Kingdom; 4 Université Paris-Saclay, Inserm, HITh, Le Kremlin-Bicêtre, France; 5 CHRU de Nancy, Nancy, France

**Keywords:** aging, coagulation, endothelial markers, naked mole rat, thrombin generation

## Abstract

**Background:**

Human aging is characterized by endothelial dysfunction that drives a systemic prothrombotic shift. In contrast, the long-lived naked mole rat (NMR) represents a unique model of delayed aging, exhibiting a notable resistance to age-related pathologies. However, while its cardiovascular stability is well-documented, the NMR hemostatic profile across its lifespan remains unexplored.

**Objectives:**

To assess whether NMRs undergo age-related hypercoagulability and to compare their hemostatic trajectory with that of humans.

**Methods:**

We compared young (2-year-old) and aged (20-year-old) NMRs. Assessments included clotting factor quantification, endothelial markers, and integrative thrombin generation assays. Plasma from human volunteers (20-year-old vs 80-year-old NMRs) were used as a reference point for typical hemostatic aging.

**Results:**

NMRs maintained cellular blood composition and showed no age-related increase in markers of endothelial activation (including von Willebrand factor, factor VIII, tissue factor pathway inhibitor, soluble thrombomodulin, and tissue plasminogen activator). While aged NMRs showed a modest increase in fibrinogen and D-dimer, this rise was significantly lower than the 2- to 5-fold elevations seen in elderly humans. Most notably, thrombin generation potential remained identical between young and aged NMRs. In contrast, humans exhibited a marked age-dependent shift toward accelerated and heightened thrombin production.

**Conclusion:**

NMRs possess the ability to bypass the pathologic clotting shifts that drive thrombotic events in humans, effectively decoupling chronologic aging from prothrombotic risk. By maintaining stable endothelial coagulation markers and an unchanged thrombin-forming capacity throughout their lifespan, NMRs appear naturally protected against age-dependent hypercoagulability.

## Essentials

• Vascular aging promotes progressive endothelial dysfunction, increasing the risk of thrombosis.
• The hemostatic profile was compared between young and aged naked mole rats (NMRs) and humans.
• NMR maintains stable plasma thrombin generation and endothelial coagulation markers as it ages.
• NMR decouples aging from prothrombotic risk, offering a model for healthy hemostatic aging.

## 1 Introduction

In humans, aging is intrinsically coupled to a prothrombotic shift, characterized by progressive endothelial dysfunction, chronic low-grade inflammation, and a heightened capacity for thrombin generation [1]. In aging, the delicate equilibrium between procoagulant drivers and anticoagulant regulatory pathways is disrupted, leading to an increased plasma concentration of coagulation factors, alongside a marked elevation in fibrin turnover markers like D-dimer [2]. This hemostatic aging significantly elevates the risk of cardiovascular and thrombotic diseases in elderly populations [3,4].

In contrast to the human aging trajectory, the naked mole rat (NMR) exhibits extraordinary longevity for a rodent model, with an average maximum lifespan of >30 years. They show notable resistance to age-related pathologies [5,6]. NMRs maintain cardiovascular health into extreme old age, showing minimal changes in cardiac structure, arterial stiffness, or vascular reactivity [7,8]. While previous research has elucidated the genomic and proteomic adaptations contributing to NMR longevity [9,10], whether NMRs maintain hemostatic balance across their lifespan remains largely unexplored. Specifically, it is unknown whether NMRs are subject to the same age-related hypercoagulability that serves as a hallmark of aging in humans. Despite existing reports on the specialized clotting mechanisms of the subterranean blind mole rat (*Spalax*) [11], the hemostatic profile of the NMR has, to our knowledge, remained unexplored likely due to the technical challenges of obtaining high-quality blood samples from this species. To address this gap, we investigated the hemostatic profile of NMRs across 2 distinct age groups (2 and 20 years) using both measurements of individual clotting factors, including endothelial coagulation markers and dynamic, integrative thrombin generation assays, to provide a comprehensive assessment of the latent procoagulant and anticoagulant forces within the plasma. To investigate species-specific protection against age-dependent hypercoagulability, we compared the age-related coagulation trajectories of NMRs and humans, both of which have a similar extended longevity relative to body size [12].

## 2 Methods

### 2.1 Animals and blood drawing

NMRs of 2 and 20 years of age were removed from social groups housed at the School of Biological and Behavioural Sciences, Queen Mary University of London [10], UK. Our study included both male and female NMRs, knowing that among nonbreeding male and female NMRs, sex differences are minimal as they have not gone through puberty due to suppression of their reproductive development by the queen [13]. Animals were maintained at 30 °C and 21% O_2_ in 50% humidity with a natural light–dark cycle in artificial burrow systems composed of interconnected perspex tubing with separate chambers for nesting and a latrine, simulating burrow conditions of their natural habitat. They were fed an ad libitum diet of a variety of chopped root vegetables, such as sweet potato and turnip, and fruits. Both sexes were used in the study. NMRs were anesthetized with pentobarbital, and whole blood samples were obtained by cardiac puncture of the right ventricle following opening of the diaphragm and left side of the thoracic cage using a 26-gauge needle into syringes containing 50 μL of citrate 3.2% reaction tubes. Depending on the final blood volume (usually between 0.5 and 1 mL), more citrate was added to reach 1:10 anticoagulant ratio.

Complete blood counts and hematocrit were determined with an automatic cell counter KX-21N (Sysmex Corporation). Blood was centrifuged at 200*g* for 10 minutes at room temperature, followed by a second centrifugation at 2000*g* for 10 minutes at room temperature. Platelet-poor plasma (PPP) was collected, snap frozen on dry ice, and kept at −80 °C until use.

A whole blood coagulation time was recorded in a KC10 coagulometer (Diagnostica Stago). A volume of 100 μL of citrated blood was incubated and mixed with 100 μL of platelet-rich plasma reagent diluted in CaCl_2_ (final concentration 37.5 μM) to reach 3 pM TF final concentration and trigger coagulation.

### 2.2 Aging human populations

The human reference population comprised 22 young adults from the TELARTA study [14] and 21 elderly participants (aged >75 years) from the ADELAHYDE-2 study [15] who had no history of cardiovascular events. Individuals with current anticoagulant therapy were excluded. These studies were approved by our institutional review board. All participants provided written informed consent.

### 2.3 Thrombin generation in NMR and human plasmas

Calibrated automated thrombography was performed at 33 °C for NMR PPP and 37 °C for human PPP in a microtiter plate fluorometer (Fluoroskan Ascent; ThermoLabsystems), using a dedicated software program (Thrombinoscope BV) [16]. Thrombin generation was triggered by 1 pM tissue factor (Innovin) and 4 μM in-house prepared phospholipids. The assay protocol for NMR PPP followed established mouse models [17,18], using a 1:6 dilution of PPP with the incubation temperature set at 33 °C to reflect the species’ physiological body temperature. Thrombin generation curves were recorded in triplicate. The thrombin generation curve yielded 5 key parameters. The following 3 were directly measured: lag time, thrombin peak, and time to peak. From these, 2 additional parameters were calculated—the velocity and the endogenous thrombin potential, which represents the area under the curve.

### 2.4 Coagulation factors

D-dimer, factor VIII (FVIII), von Willebrand factor (VWF) antigen, tissue factor pathway inhibitor (TFPI), and soluble endothelial protein C receptor (EPCR) levels were quantified in PPP by ELISA (Asserachrom; Diagnostica Stago). TFPI activity was assessed using the ACTICHROME TFPI, activity assay (CRYOPEP). Thrombomodulin (TM), tissue-type plasminogen activator (t-PA), and plasminogen activator inhibitor (PAI)-1 were quantified by ELISA (Quantikine ELISA kits and DuoSet; R&D Systems). Fibrinogen was measured in PPP samples using the TriniCLOT Fibrinogen kit (Diagnostica Stago). Quantification was limited to a subset of 2-year-old NMRs, as this assay requires a larger sample volume than other markers. Because human standards were used for the calibration of most assays, NMR results are expressed as a percentage of the 2-year-old group means, except for fibrinogen, D-dimer, and TFPI activity. To validate consistency with human assay standards, NMR samples were prepared at 2 dilution levels, confirming that all values paralleled the established calibration curves.

### 2.5 Statistical analysis

Results are presented as mean ± standard deviation. Normality was assessed using the Shapiro-Wilk test. For two-group comparisons, the Mann–Whitney *U*-test was used for non-parametric data and the t-test for parametric data. A *P* value <0.05 was considered statistically significant.

## 3 Results and Discussion

To assess age-related changes in NMR clotting mechanisms, we compared the coagulation profiles of young (2-year-old) and aged (20-year-old) animals. Due to methodological requirements and limited animal availability, it was challenging to achieve a sample size for the 20-year-old group comparable with that of the 2-year-old animals. Initial analysis of complete blood counts showed no significant changes with age (Table 1). A stable peripheral blood composition was previously reported until 12 year-old [19]. Our study thus validates and extends these observations in significantly older NMRs. The absolute lower values obtained in this study, relative to previously reported data [20], are likely explained by the citrate dilution inherent in blood drawing protocols dedicated to coagulation measurements. These findings suggest that cellular composition does not drive age-dependent changes in whole blood coagulation. This was further supported by our assay, which measured mean whole blood coagulation times of 112 ± 21 seconds for young NMRs compared with 93 ± 15 seconds for aged NMRs (*P* = .21).

**Table 1:** Complete blood count.

Parameter	2-y-old NMR	20-y-old NMR
*n*	7	4
Weight (g)	22 ± 5	38 ± 4^a^
White blood cells (×10^3^/μL)	7.9 ± 4.4	5.4 ± 1.2
Red blood cells (×10^6^/μL)	6.7 ± 0.9	6.0 ± 1.8
Hemoglobin (g/dL)	12.2 ± 2.0	11.6 ± 2.0
Hematocrit (%)	38.6 ± 5.7	34.4 ± 10.4
Mean corpuscular volume (fL)	57.8 ± 2.9	57.0 ± 0.9
Mean corpuscular hemoglobin (pg)	18.2 ± 1.8	19.9 ± 2.9
Mean corpuscular hemoglobin concentration (g/dL)	31.6 ± 3.9	34.9 ± 5.1
Red cell distribution width (fL)	38.7 ± 5.4	35.0 ± 3.1
Platelets (×10^3^/μL)	404 ± 141	381 ± 100
Mean platelet volume (fL)	6.6 ± 1.0	6.1 ± 0.5

NMR, naked mole rat.

a *P* < .05.

**Figure 1:**
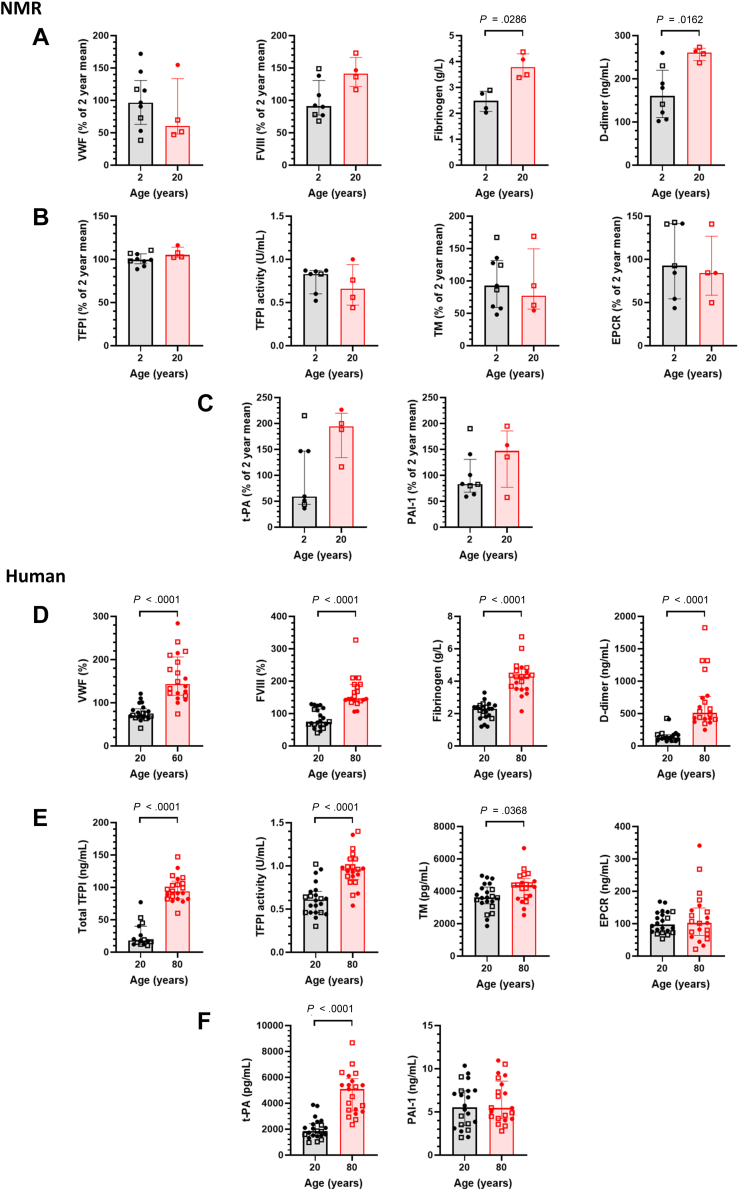
Plasma coagulation factors. (A) Procoagulant factors in naked mole rat (NMR) plasma (*n* = 7–9 and *n* = 4 for 2-year-old and 20-year-old NMRs, respectively). (B) Anticoagulant factors in NMR plasmas. (C) Fibrinolytic factors in NMR plasmas. (D) Procoagulant factors in human plasma (*n* = 22 and *n* = 21 for 20-year-old and 80-year-old humans, respectively). (E) Anticoagulant factors in human plasmas. (F) Fibrinolytic factors in human plasmas. Data are presented as medians and IQRs. Data points are categorized by sex, indicated by closed circles (males) and open squares (females). Groups were analyzed statistically by the Mann–Whitney test. EPCR, endothelial protein C receptor; FVIII, factor VIII; TFPI, tissue factor pathway inhibitor; TM, thrombomodulin; t-PA, tissue-type plasminogen activator; VWF, von Willebrand factor.

To evaluate how aging impacts the hemostatic balance in NMRs, we quantified a comprehensive panel of procoagulant, anticoagulant factors and fibrinolytic markers, mostly of endothelial origin. While age-associated endothelial cell activation typically triggers the release of factors that modulate coagulation and fibrinolysis, we found no age-related increase in NMR levels of VWF, FVIII, TFPI, soluble TM, soluble EPCR, t-PA, or PAI-1 (Figure 1A–C). While these markers exhibited some variability, the data distributions for both age groups overlapped entirely. These new results are consistent with the reported stability in endothelial function, NO production, or reactive oxygen species levels in aging (12-year-old) NMRs [7]. In contrast, both fibrinogen and D-dimer levels were significantly elevated in aged NMRs (Figure 1A). The positive correlation between these markers suggests an accelerated fibrin turnover, occurring despite the stability of endothelial markers. This increase in fibrinogen may account for the nonsignificant trend toward shorter whole blood coagulation times observed in aged animals.

To provide a clinical reference point, we use a validated population of elderly human individuals free from cardiovascular complications or prior thromboembolic events. We measured identical parameters in young (20 year-old; 15 men and 7 women) and elderly (80 year-old; 9 men and 12 women) human subjects since human aging is characterized by a well-documented prothrombotic shift [1]. Human VWF, FVIII, TFPI, soluble TM, and t-PA increased with age (Figure 1D–F). Although previous studies have reported elevated plasma levels of PAI-1 and EPCR in individuals >35 or 45 years of age [21,22], our data showed no significant differences across age groups (Figure 1E). This discrepancy may stem from the higher proportion of men in our younger group. Because men typically exhibit higher circulating concentrations of PAI-1 and EPCR than women, their overrepresentation in the younger group potentially offset the differences between the 2 age groups. In line with the well-documented age-related shift in the fibrinogen–fibrin axis, fibrinogen and D-dimer concentrations were markedly higher in the elderly group. Notably, independent of sex, humans exhibited a much more substantial age-related elevation in fibrinogen and D-dimer levels (2-fold and 5-fold increases, respectively) than NMRs, where these markers rose by <1.5-fold.

Collectively, these data indicate that NMRs maintain a remarkably stable endothelial profile between 2 and 20 years of age. This profile deviates clearly from the age-related changes seen in humans, which is characterized by a systemic increase in endothelial activation markers and an important 2- to 5-fold rise in fibrin turnover. Although fibrin turnover in NMRs shows a slight age-related acceleration, it appears to occur independently of the widespread endothelial dysfunction that typically drives thrombotic risk in humans.

**Figure 2:**
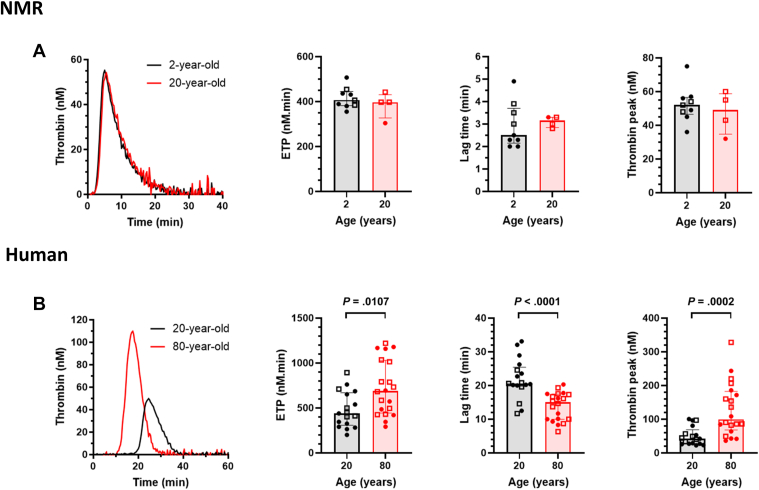
Thrombin generation in platelet-poor plasma (PPP). (A) Representative thrombin generation curves and parameters in PPP from naked mole rats (NMRs). (B) Representative thrombin generation curves and parameters in PPP from human. Endogenous thrombin potential (ETP) values were calculated as area under the curves of thrombin generation. Lag time and thrombin peak were derived from thrombin generation curves. Data are presented as medians and IQRs. Data points are categorized by sex, indicated by closed circles (males) and open squares (females). Groups were analyzed statistically by the Mann–Whitney test.

To move beyond individual factors that fail to account for the inherent complexity of the coagulation cascade, we used an integrative thrombin generation assay in PPP. This approach provides a more comprehensive assessment of how aging influences the overall balance between procoagulant and anticoagulant pathways. As established tools for the global assessment of hemostasis, thrombin generation assays are instrumental in diagnosing and tailoring treatment for various inherited and acquired bleeding and thrombotic disorders [23,24]. Thrombin generation profiles remained stable across age groups in NMRs, with aged NMRs exhibiting curves indistinguishable from those of young animals (Figure 2A). Specifically, the total endogenous thrombin potential, the kinetics of thrombin generation (lag time, time to peak, and velocity), and the maximum thrombin concentration (peak height) remained unchanged with aging (Table 2 and Figure 2A). In contrast, and consistent with previous reports [25,26], analysis of thrombin generation in humans revealed a marked age-dependent shift toward accelerated and heightened thrombin production (Table 2 and Figure 2B). Thrombin generation assays and D-dimer quantification are widely used for hypercoagulability testing [27]. This dual-method approach is particularly informative because the 2 markers reflect different biological processes. While D-dimer serves as an acute-phase reactant and a marker of prior *in vivo* fibrin turnover, thrombin generation assays evaluate the latent hemostatic potential of the plasma. By combining these methods, we could distinguish between ongoing fibrinolytic activity and the intrinsic capacity of the coagulation cascade. Our data revealed that while NMRs exhibit a slight age-related increase in fibrin turnover, their underlying thrombin-forming potential remains remarkably stable, unlike the accelerated profiles observed in aging humans. Endothelial coagulation and fibrinolytic markers remain stable in 20-year-old NMRs but are significantly elevated in older humans; these findings align with the stable and increased thrombin generation profiles observed in each species, respectively.

**Table 2:** Thrombin generation parameters.

Parameter	2-y-old NMR	20-y-old NMR	20-y-old humans	80-y-old humans
*n*	9	4	17	20
ETP (nM × min)	416 ± 46	385 ± 58	480 ± 199	726 ± 306^a^
Lag time (min)	2.0 ± 1.0	3.1 ± 0.3	21.9 ± 6.0	13.8 ± 4.2^a^
Peak (nM)	53 ± 11	48 ± 13	50 ± 27	129 ± 79^a^
Time to peak (min)	5.6 ± 1.7	5.9 ± 0.7	26.2 ± 6.3	17.4 ± 4.5^a^
Velocity (nM/min)	22 ± 8	18 ± 7	12 ± 8	44 ± 38^a^

ETP, endogenous thrombin potential; NMR, naked mole rat.

a *P* < .05.

In conclusion, NMRs preserve their global hemostatic balance throughout mid-to-late life. It is difficult to establish a precise human age equivalent for the NMR; however, since 20 years exceeds two-thirds of their maximum lifespan, it serves as a robust proxy for advanced age. By maintaining stable endothelial function and an unchanged thrombin potential, NMRs appear to be naturally protected against the age-dependent hypercoagulability that drives thrombotic events in aging humans. The slight elevation in D-dimer levels, in the absence of accelerated thrombin generation kinetics, suggest an alternative pathway of fibrin turnover rather than a pathologic precursor to thrombosis. Such hemostatic resilience aligns with the reported lack of cardiovascular pathology in NMRs, despite the fact that atherothrombosis remains unexplored in this species [8,10,28,29]. Our study demonstrates that the NMR serves as a robust model for analyzing the relationship between the endothelium and age-related coagulation shifts [5]. Contrasting the NMR findings with human data highlights a fundamental divergence in vascular lifespan trajectory, suggesting that NMRs have developed specific endothelium adaptation to decouple chronological aging from prothrombotic risk. A better understanding of the underlying mechanisms may be of interest to identify potential key actionable targets.
